# Polyorromenitis

**Published:** 1900-12-29

**Authors:** 


### POLYORROMENITIS.

Objectionable as it is to combine Greek and Latin in the compounding of new names, we cannot but feel that the word "polyserositis" is both better sounding and more self-explanatory, as indicating an inflammation of several serous membranes, than the word "polyorromenitis," notwithstanding the etymological correctness of the latter. It appears that *ὀρρός* in Greek means serum, and that when we wish to speak of a multiple inflammation of serous membranes polyorromenitis is the word to use. It is an uncouth term, but there it is, and as it is accepted by Dr. Frederick Taylor,[1] who has described the condition to which it is applied, we must make the best of it.

Dr. Taylor says that the frequent association of serous cavities in a common lesion is a fact which has long attracted attention, not only in the case of combined tuberculous peritonitis and pleurisy, but also in the case of some more acute diseases, such as acute rheumatism—which often invades the pericardium and pleura simultaneously—and pyæmia which may attack every one of the four great serous sacs at the same time. It is clear, however, that we must take care not to regard this conjunction of events as a separate disease, and that we must not think we have got to the bottom of the matter when we have given it this imposing name, for it only represents a common grouping of pathological conditions. Still, this grouping is of such frequency that we may properly ask what are the diseases, the bacteria, or the toxins which will cause it.

As regards origin it may be said that acute polyorromenitis may be caused by the pneumococcus, as we see often enough in pneumonia, in the course of which both pericarditis and pleurisy may occur; another cause of the acute disease is streptococcal or staphylococcal invasion, as in pyæmia and septicæmia; another is acute rheumatism; and in a small number of cases the acute disease may be set up by tuberculosis. Tuberculosis, however, plays its most important part in the production of the subacute and chronic forms of the disease, of which it is by far the most frequent cause. The subacute or chronic forms usually begin in one cavity and subsequently invade the others, by far the most frequent place for commencement being the peritoneum. Italian observers have gone so far as to maintain that subacute and chronic polyorromenitis is always tuberculous; but Dr. Taylor, while assenting to the general proposition that such cases are as a rule tuberculous, holds that they need not always be so, and he points on the one hand to the cases that recover, especially in children, without any further sign of tuberculous infection, and on the other to some cases which die with dense and thick adhesions of several cavities, and yet without any post-mortem sign of tuberculosis.

[1] Brit. Med. Jour., Dec. 15.

